# A single-centre, retrospective study on the impact of omitting preoperative antibiotic prophylaxis on wound infections in minor orthopedic implant removals

**DOI:** 10.1007/s00068-025-02769-y

**Published:** 2025-02-07

**Authors:** Cyrill Pfammatter, Jan Hambrecht, Yannik Kalbas, Valentin Neuhaus, Christian Hierholzer, Claudio Canal

**Affiliations:** 1 Department of Traumatology, University Hospital Zurich, Zurich, 8091 Switzerland; 2 Department of Surgical Research, Harald Tscherne Laboratory for Orthopedic and Trauma Research, University Hospital Zurich, Zurich, 8091 Switzerland; 3 Department of Surgery, Cantonal Hospital Thurgau, Pfaffenholzstrasse 4, Frauenfeld, 8501 Switzerland

**Keywords:** Implant removal, Wound infection, Preoperative antibiotic prophylaxis

## Abstract

**Background:**

The use of preoperative antibiotic prophylaxis (POAP) in elective implant removal (IR) is controversial due to a lack of evidence-based recommendations. First-generation cephalosporins, which are commonly used in orthopedic IR, are believed to reduce wound infection risks. However, the potential for serious side effects had raised concerns about their necessity. This study was intended to evaluate whether omitting POAP in small IR increases the risk of wound infections.

**Methods:**

This retrospective, single-centre cohort study was conducted at a level I trauma centre in Switzerland, including patients who underwent IR between January 1, 2016, and December 31, 2021. The IR procedures involved the upper extremities (UEs), such as the clavicle, olecranon, radius and ulna, as well as the lower extremities (LEs), such as the patella, tibia, fibula, (bi)malleolar and foot. Postoperative follow-up included clinical and radiological evaluations 6 weeks after surgery. The outcomes assessed were deep wound infections, wound healing complications, refractures, persistent pain, bleeding, neurovascular injuries and muscle hernias.

**Results:**

Of the 273 patients (mean age: 42.1 ± 14.5; 44% female), 117 (42.9%) received POAP. In the LE group (*n* = 141), 51.1% received POAP; in the UE group (*n* = 132), 34.1% received POAP. Eleven (4.0%) wound-healing disorders were documented, with five (4.3%) in the POAP group and six (3.8%) in the non-POAP group (*p* = 1). No deep wound infections were observed.

**Conclusion:**

Withholding POAP in elective IR procedures does not significantly increase wound infection rates, suggesting it may be unnecessary in uncomplicated cases.

## Introduction

Elective implant removal (IR) is common in orthopedic surgery in industrialized countries, being performed in up to 30% of operations [1]. These operations are seen as a standard procedure; however, there are few clinical studies showing their relevance and benefit [2]. Although there are clinical guidelines regarding the indications for IR, there is still a lack of evidence-based recommendations in this regard [3]. Therefore, the decision to perform IR is highly individual and based on different indications. Patients who desire IR either show absolute indications, such as infections and non-unions, or relative indications, such as symptomatic relief; the restriction of movement and nonspecific and, sometimes, weather-dependent symptoms of discomfort. For the latter, the benefits of IR have not been consistent [4].

Postoperative complications depend on the location and type of the implant removed, and they are estimated to range from 11 to 21% [5]. Studies investigating the outcomes of hardware removal in a specific anatomical region have shown a wide range of rates for overall and specific complications [6, 7]. One of the most common complications is wound infection. Its incidence varies with the duration of surgery, the size of the surgical incision, diabetes and smoking status and other patient risk factors [8]. In the literature, an incidence rate for wound infection of up to 11% has been described [9]. Preoperative antibiotic prophylaxis (POAP) has been hypothesised to reduce incidence rates, but its clinical relevance has not yet been established. Some guidelines include POAP, but these mention its contradictory position regarding IR [10]. A 2017 study of elective IR demonstrated a modest absolute risk reduction of 1.7% for the therapy group [11]. Currently, while regional guidelines exist, there are no international standards directing the use of POAP in IR. Consequently, the implementation of POAP largely varies according to individual surgeons’ discretion [12].

Antibiotic administration can lead to adverse effects, including allergic reactions, gastrointestinal disturbances, dermatological reactions, edema and headaches, which result in higher cost and lower quality of life [13]. One of the most prominent concerns arising from antibiotic overuse is the development of multidrug-resistant bacteria. Globally speaking, the Lancet estimated 4.95 million deaths in 2022 associated with antimicrobial resistance [14].This raises a pertinent question regarding the necessity of antibiotics in IR. Therefore, the aim of this study was to explore the feasibility of omitting POAP in elective same-day surgeries for small IR in a single level I trauma centre given the possibility of wound infections.

## Materials and methods

### Study design

This research constitutes a retrospective, single-centre, cohort study performed at a level I trauma centre, and its documentation is aligned with the guidelines outlined in the STROBE (strengthening the reporting of observational studies in epidemiology) statement [15].

### Ethical consideration

This investigation obtained approval from the cantonal ethics committee and was executed in strict adherence with the principles delineated in the Declaration of Helsinki (PB_2016_01888). Only patients who provided written consent were included.

### Study population

Patients undergoing elective IR procedures on the upper and lower extremities between the 1 January 2016 and 31 December 2021 at the outpatient facility of a Swiss level I trauma centre were enrolled in this study. We registered all patients who presented to our outpatient clinic with a non-infected and radiologically consolidated fracture of the upper or lower extremities following open or closed reduction and internal fixation and were requesting IR. To facilitate meaningful comparisons, we chose to focus our cohort on the most frequently encountered anatomical regions for IR. These include the clavicula, olecranon, ulna, radius, patella, tibia, fibula, ankle and foot. Patients who underwent elective IR in other anatomical regions (e.g., the humerus, femur, and sacrum) were excluded from the study. Additionally, patients with Kirschner wires only, intramedullary nails, large plates (more than 10 holes), tension band wiring only and external fixators only were excluded as well. Furthermore, patients under 18 years of age; pregnant patients; patients lacking written informed consent; individuals with pre-existing infection; and patients with non-union and individuals with incomplete data, such as a missing initial operation report or missing follow-ups, were excluded from participation in this study.

### Surgery and aftercare

All patients manifested radiologically consolidated fractures after open or closed reduction and internal fixation procedures were performed on either the upper or lower extremities, with a postoperative interval of at least 1 year for distal radius/ulna and olecranon fractures and typically at least 2 years for clavicle fractures. The choice between general anesthesia and regional anesthesia was determined based on the individual preferences of the patients. All patients underwent elective same-day surgery in an outpatient setting. The decision to administer preoperative antibiotic prophylaxis was based on the surgeons’ preferences. The follow-up protocol included a wound assessment 1 day postoperatively and a clinical examination with radiological evaluation 6 weeks postoperatively. In the interim period, wound care and monitoring were managed by the patients’ general practitioners or a home healthcare organization, depending on their individual capacity to manage the wound effectively.

### Clinical evaluation and outcomes

Patients were examined before and after surgery. At the clinical examination and radiological control 6 weeks postoperatively, complications such as deep wound infection, wound-healing disorder, refracture, persistent bleeding, neurovascular injury, persistent pain, muscle hernia and re-hospitalisation were identified. Our primary outcomes to be assessed were deep wound infection and wound-healing disorder, examined specifically in association with the omission of POAP. The secondary outcome included the previously mentioned complications. We compared the rate of complications in the upper extremity (UE) and lower extremity (LE) groups and we investigated the association between complication and comorbidities and the demographics as well as the perioperative variables of our cohort. Of the patients receiving POAP, 98.3% were administered Cefazolin, typically at a single-shot dosage of 2 g.

### Definitions

Deep wound infection was defined as redness, swelling and heat around the surgical incision; localised increased pain and tenderness; pus or other drainage from the wound; fever or systemic symptoms (general malaise or chills) and a foul odour from the wound. Deep wound infection was diagnosed based on a clinical examination and blood tests with elevated infection parameters (c-reactive protein and white blood cell count). Microbiological evidence was collected whenever possible, using either swabs or tissue samples, to confirm the presence of infection and identify the causative organism. However, specific anatomo-pathological were not applied in our diagnostic process. Wound-healing disorder was defined as a wound that was not fully closed after 6 weeks. Refracture was defined as a fracture of the bone from which the implant was removed. The fracture had to be visible on X-ray. Persistent bleeding was defined as postoperative bleeding lasting more than 2 days. Neurovascular injury was defined as the onset of postoperative paresthesia and/or the onset of inadequate arterial perfusion in the distal region. Muscle hernia was defined as a postoperative newly protruding muscle in the region of the surgical incision. Persistent pain was defined as the patient’s subjective perception of pain that lasted for more than 4 weeks. Partial IR was defined as IR in which certain components were intentionally left in place according to the treatment plan (e.g., in bimalleolar stabilization, in which only the medial material was taken out). Remaining material was defined as hardware being unintentionally retained due to breakage or other intraoperative complications.

### Statistics

Statistical analyses were performed using R software [R], specifically R Core Team (2024) (R: A language and environment for statistical computing (Version 4.2). Retrieved June 18, 2024, from https://www.r-project.org/. Continuous data were expressed as means ± standard deviations, whereas categorical variables were expressed as counts and percentages. The normality of the data was assessed visually using histograms. For parametric data, unpaired student’s t-tests were used. Non-parametric data were analysed using Wilcoxon–Mann–Whitney tests. Binary categorical data were analysed using Fisher’s exact test. Odds ratios and 95% confidence intervals were estimated using conditional maximum likelihood. For non-binary categorical data, the Chi-squared test with Yates’s correction for continuity was used. Statistical significance was defined as a p-value < 0.05.

## Results

### Study population

**Fig. 1:**
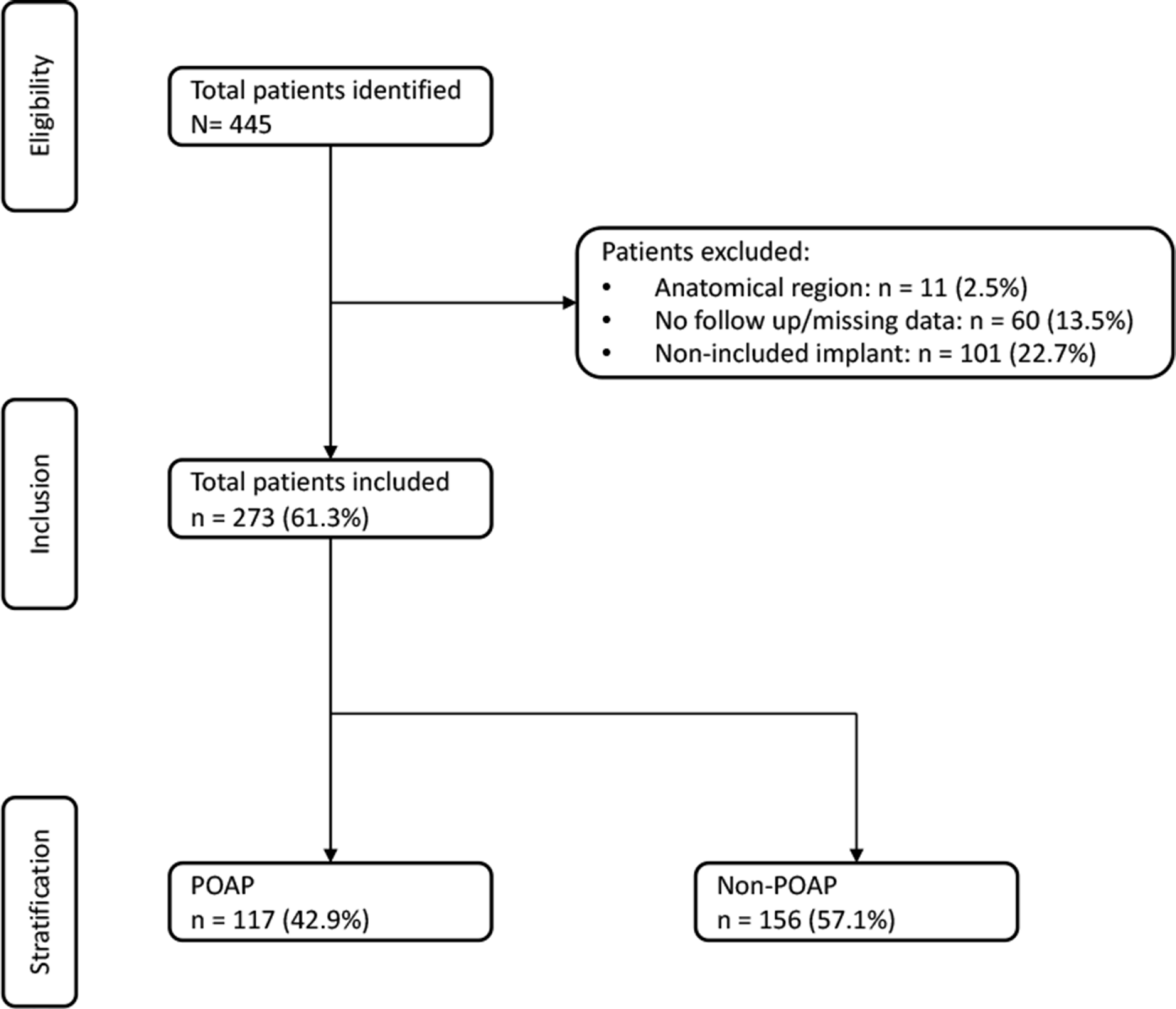
Patient selection flowchart

We enrolled a total of 445 patients in this study, of whom 273 met our inclusion criteria. We excluded 60 patients (13.5%) due to incomplete data in their reports, 11 patients (2.5%) due to the anatomical region of the IR and 101 patients (22.7%) due to the kind of implant used (Kirschner wires, intramedullary nails, large plates, tension band wiring and external fixators). Finally, we were able to include 117 patients who underwent IR with POAP and 156 patients who underwent IR without POAP (Fig. 1). When categorized by anatomical region, we recorded 132 cases in the UE: 55 clavicle, 23 olecranon, 46 radius, and 8 ulna IR cases. In the LE, there were 141 cases in total: 36 tibia, 51 fibula, 31 ankle, 17 foot, and 6 patella IR cases.

### Patient demographics and procedural parameters

Of the 273 included patients, 153 (56%) were male, and 120 (44%) were female, with a mean age of 41.2 years (range: 17–82 years, SD = 14.5). Eighty-seven (31.9%) patients were suffering from comorbidities. The most frequently encountered comorbidities that were relevant for infections and wound-healing disorder were cardiovascular diseases (40.2%), followed by allergies (40.2%), skeletal disorders (12.6%) and metabolic diseases (4.6%). The UE and LE groups had some demographic differences (Table 1). Smoking was seen in 70 patients (25.6%). The number of smokers in the LE group was almost twice as high as in the UE group.

**Table 1:** Patient demographics

	Overall	Upper extremity	Lower extremity	*p*-value
N	273	132	141	
**Demographics**				
Sex = F (%)	120 (44.0)	57 (43.2)	63 (44.7)	0.899
Mean age in years (SD)	41.2 (14.5)	41.3 (14.7)	41.0 (14.3)	0.866
Comorbidities = Yes (%)	87 (31.9)	39 (29.5)	48 (34.0)	0.505
Smoking = Yes (%)	70 (25.6)	24 (18.2)	46 (32.6)	0.01

n number, F female, SD standard deviation

### Perioperative variables

The mean duration of surgery was 32.8 min (range: 3–165 min, SD = 18.7). The mean surgical duration of IR in the UE group was over 10 min longer than in the LE group.

In the UE group, regional anesthesia was used four times more often. Another difference was the application of preoperative antibiotic prophylaxis, which was based on the surgeons’ preferences. In the UE group, 45 (34.1%) patients received POAP, as compared to 72 (51.1%) in the LE group. Consequently, doctors were 2.01 times more likely to use POAP in the lower extremities. Partial IR was performed in 38.6% in the LE group and in 7.6% in the UE group (Table 2).

**Table 2:** Perioperative variables

	Overall	Upper extremity	Lower extremity	*p*-value
**Perioperative variables**				
Type of anesthesia = regional (%)	33 (12.1)	27 (20.5)	6 (4.3)	< 0.001
POAP = Yes (%)	117 (42.9)	45 (34.1)	72 (51.1)	0.007
Partial IR = Yes (%)	64 (23.5)	10 (7.6)	54 (38.6)	< 0.001
Mean duration of surgery in minutes (SD)	32.8 (18.7)	38.7 (15.8)	27.4 (19.7)	< 0.001

### Postoperative outcome

In the UE group, nine patients (6.8%) experienced persistent pain, one (0.8%) had persistent bleeding, three (2.3%) developed wound-healing disorders, one (0.8%) presented with a muscle hernia and three (2.3%) suffered from refractures; however, no neurovascular injuries were observed. All refractures occurred in the clavicle, resulting in a clavicle refracture rate of 5.5%. In the LE group, 17 patients (12.1%) reported persistent pain, five (3.5%) experienced persistent bleeding, eight (5.7%) had wound-healing disorders, there were no cases of muscle hernia and there was one (0.7%) case of neurovascular injury and no refractures (Table 3).

**Table 3:** Postoperative outcome

	Overall	Upper extremity	Lower extremity	*p*-value
**Postoperative Outcome**				
Complication = Yes (%)	21 (7.7)	8 (6.1)	13 (9.2)	0.452
Wound-healing disorder = Yes (%)	11 (4.0)	3 (2.3)	8 (5.7)	0.263
Persistent bleeding = Yes (%)	6 (2.2)	1 (0.8)	5 (3.5)	0.247
Persistent pain = Yes (%)	26 (9.5)	9 (6.8)	17 (12.1)	0.205
Muscle hernia = Yes (%)	1 (0.4)	1 (0.8)	0 (0.0)	
Neurovascular injury = Yes (%)	1 (0.4)	0 (0.0)	1 (0.7)	
Refracture = Yes (%)	3 (1.1)	3 (2.3)	0 (0.0)	0.223

A 10% lower rate of comorbidities relevant for wound healing in patients who received POAP can be detected as compared to those who did not (*p* = 0.08). The distributions of sex, age and smoking status were similar between the two groups. The duration of surgery and the rates of wound-healing disorder and refracture were also comparable in both groups. The postoperative outcomes in the POAP and non-POAP groups only differed by more than 1% for persistent pain, with 12.8% of patients in the POAP group suffering from persistent pain, as compared to 7.1% in the non-POAP group (*p* = 0.16). No patients in either the POAP or the non-POAP group developed deep wound infections. In the POAP group, five patients (4.3%) suffered from a wound-healing disorder, while six patients (3.8%) in the non-POAP group did (*p* = 1). Other factors, such as smoking and comorbidities, were not associated with wound issues. Persistent bleeding was present in 3.2% in the POAP group compared to 0.9% in the group without POAP (Table 4).

**Table 4:** Primary outcome

	Overall	Non-POAP	POAP	*p*-value
Sex = F (%)	120 (44.0)	67 (42.9)	53 (45.3)	0.792
Mean age in years (SD)	41.2 (14.5)	39.46 (13.57)	43.48 (15.47)	0.023
Smoking = Yes (%)	70 (25.6)	39 (25.0)	31 (26.5)	0.889
Mean duration of surgery in minutes (SD)	32.8 (18.7)	30.85 (14.30)	35.49 (23.20)	0.043
Comorbidities = Yes (%)	87 (31.9)	57 (36.5)	30 (25.6)	0.075
Wound-healing disorder = Yes (%)	11 (4.0)	6 (3.8)	5 (4.3)	1
Refracture = Yes (%)	3 (1.1)	2 (1.3)	1 (0.9)	1
Persistent pain = Yes (%)	26 (9.5)	11 (7.1)	15 (12.8)	0.162
Deep wound infection = No (%)	273 (100.0)	156 (100.0)	117 (100.0)	
Persistent bleeding = Yes (%)	6 (2.2)	5 (3.2)	1 (0.9)	0.371

### Secondary outcomes

We found that in 31.0% of the operations performed by residents, POAP was used. In comparison, senior consultants used POAP in 79.1% of the operations. Thus, senior consultants were more likely to use POAP (odds ratio: 8.32). The mean duration of surgery led by senior consultants was 36.9 min, while that of residents was 31.5 min. Both groups performed surgery on the lower extremities in approximately half of cases and exhibited similar rates of partial IR post-surgery. However, senior consultants were 2.5 times more likely to leave remaining material in the surgical site.

The number of partial IR in patients without POAP was 24 (15.4%), as compared to 40 (34.5%) in the POAP group. Doctors were 2.88 times more likely to use POAP in partial IR.

## Discussion

Preoperative antibiotic prophylaxis is commonly used in IR to prevent wound infections, although its use remains controversial. Studies have yielded conflicting evidence regarding the effectiveness of prophylaxis [11, 12, 16]. First-generation cephalosporins are commonly used for this prophylaxis, as they effectively penetrate bone and muscle [17, 18]. While many studies have focused on comparing various antibiotics for prophylactic use in orthopedics [19, 20], few studies have specifically addressed whether such prophylaxis should be routinely used, especially in IR. The side effects of antibiotics can be serious, ranging from skin rash, eosinophilia and diarrhea to acute kidney damage. Dermatologic reactions occurred in 1 to 5% of cases, eosinophilia in 3 to 10%, leukopenia in 1 to 2%, diarrhea in 1 to 10% and kidney damage in 8 to 13%, as reported in various studies [17, 21–23]. Thus, our aim was to determine whether the withholding of POAP in elective, simple IR significantly increases wound infections as compared to elective IR with POAP.

Our key results are as follows:

- POAP does not have a significant effect on deep wound infections and wound-healing disorders when used for patients with minor IR.
- The use of POAP was significantly higher in the LE group as compared to the UE group.

Other studies have reported wound infection rates for IR ranging from 3.0% [24] to 14.9% [9, 11]. These findings are consistent with our results, which showed an overall wound infection rate of 4.0%. Specifically, wound-healing disorders accounted for 4.0% of cases, while deep wound infections were not observed.The infection rate is influenced by various factors, such as patient comorbidities, smoking status, age and the duration of surgery [25, 26]. Additionally, factors regarding hygiene in and the sterility of the operation room also play crucial roles in determining the risk of postoperative infections [27].

Our results show a low percentage of infections (4.0%), which may be attributed to the high standard of equipment, sound operating room protocols and the strict hygiene measures of the surgical team. Another factor could be the careful selection of patients, as multimorbid patients were not included in our study, as they were operated in another setting. Additionally, we excluded cases involving intramedullary nails, large plates and external fixators, focusing exclusively on small IR, as these patients were managed in an inpatient setting and subject to a different billing process. This selection criterion may contribute to the low infection rate observed in our study.

Regarding our primary objective of assessing wound infections, with a specific focus on their association with the omission of POAP, Redais et al. [24] conducted a study involving 965 patients to evaluate the efficiency of antibiotic prophylaxis in preventing surgical site infection following IR. They found a significant difference in infection rates: the group that received POAP had an infection rate of 3.0%, as compared to 11.1% in the group without POAP. In contrast, Backes et al. [11], who included 477 patients in their study, did not observe a significant difference between groups, with infection rates of 13.2% for those who underwent POAP and 14.0% for those who did not. Few randomised controlled trials address this topic, as demonstrated by Rather et al. [28].

Our study results align with the findings of Backes et al. [11], revealing a wound infection rate of 4.3% in the POAP group and 3.8% in the group without POAP. This suggests that while POAP may slightly reduce infection rates, the difference observed in our study is not substantial enough to establish a significant impact. These findings contribute to the ongoing debate on the effectiveness of prophylactic antibiotics in preventing wound infections post-surgery, highlighting the need for further research to establish clear guidelines.

Regarding our secondary outcome, we examined the use of POAP based on the skill and experience of the operating surgeon, comparing residents to senior consultants. Residents used POAP in 31.0% of their operations, whereas senior consultants did so in 79.1% of cases. We found that senior consultants were more likely to use POAP. The senior consultants may have been handling more complex IRs due to their higher skill and experience levels, necessitating the use of POAP as a safety measure. This complexity is suggested by our data, as senior consultants led more surgeries with retained screws and had longer operation durations on average. However, other factors contributing to this complexity could not be identified in our analysis. To the best of our knowledge, we have not identified any studies conducted on this specific topic.

Examining the use of POAP in patients with partial IR, we observed a significantly higher rate of POAP administration. Surgeons were 2.9 times more likely to use POAP in cases in which the implants were only partially removed (p-value < 0.001). Furthermore, when the patient was older and the operation lasted longer, surgeons were more likely to use POAP. These findings, in conjunction with the higher likelihood of POAP use among senior consultants, suggest that these operations were more complex, thereby necessitating the use of POAP. When comparing the administration of POAP in the UE and LE groups, we found that in the UE group, 34.1% of patients received POAP, while in LE group, 51.1% of patients did. Therefore, surgeons were twice as likely to use POAP in IR for the LE group.

While scientific literature does not cover this area specifically, it describes wound infection and complication rates in the context of the LEs and UEs [9, 11, 29]. Regarding IR, Backes et al. [9] found wound infection rates of 5.5% in the UEs and 12.5% in the LEs. Dong et al. [30] reported a 7.3% wound infection rate in the UEs, while Backes et al. [11] found a 14.0% wound infection rate in the LEs. Our findings show a 2.3% wound infection rate in the UEs and a 5.7% rate in the LEs, which are lower than those in the recent literature but suggest the same trend.

When examining the higher rate of wound infections in the LEs, several factors may contribute: thin layers of tissue, greater strain on the skin, challenges related to rest and activity and enhanced blood circulation due to exercise aids during regeneration [31, 32]. However, pain may prevent patients from walking and, consequently, engaging in exercise. Additionally, many patients suffer from chronic venous insufficiency, which impairs functional blood circulation and, thus, may hinder wound healing, potentially increasing the risk of infection [33]. This may explain why POAP was used more frequently in the LEs. Overall, our findings indicate that the elective IR of small plates is a safe procedure and produces few wound infections with or without POAP.

### Strengths

In this study, we were able to include a large sample with relatively large subgroups for the UEs and LEs. The same goes for the various subgroups of the UE and LE groups. In our sample, the homogeneity of the patients was high. Furthermore, the data were collected during a standardised follow-up period of 6 weeks postoperatively, ensuring consistency in outcome assessments. The follow-up evaluations were conducted by experienced trauma surgeons, which enhanced the quality and accuracy of the clinical assessment and documentation.

### Limitations

Several limitations must be acknowledged. The admission of POAP was depending on the surgeons’ preference, which is a strong limitation. Additionally, this is a retrospective, single-centre, cohort study, which inherently carries the risk of selection bias. Notably, multimorbid patients were operated on an in-hospital setting, potentially leading to the underestimation of infection and overall complication rates. Moreover, because documentation beyond 6 weeks was not collected, complications arising later were not detected. Additionally, the single-centre design prevents the generalisation of our findings to other institutions with differing patient demographics and clinical practices.

## Conclusion

When analysing patients with uncomplicated IR, we found in this study that the use of POAP has no significant impact on the incidence of deep wound infections or wound-healing disorders. Therefore, the administration of POAP should not be routine but, rather, carefully considered based on the anticipated complexity of an IR procedure. This supports the recommendation to discontinue the use of POAP in cases where IR is anticipated to be straightforward. Particularly in LE procedures, in which our data and similar research indicate high infection rates, it is crucial to thoroughly assess the risk of infection. Factors such as the expected complexity of a surgery, the anatomical site involved and the presence of comorbidities must be evaluated before deciding whether to administer POAP.

## Data Availability

No datasets were generated or analysed during the current study.

## Abbreviations

IR: Implant removal UE Upper extremity
POAP: Preoperative antibiotic prophylaxis LE Lower extremity
STROBE: Strengthening the reporting of observational studies in epidemiology
SD: Standard deviation

## Declarations

### Declaration of AI-assisted technologies in the writing process

During the preparation of this work, the authors used ChatGPT (OpenAI. (2024) ChatGPT (Version 4) (AI model)). The text was retrieved on August 19, 2024, from https://www.openai.com/chatgpt). The authors also used DeepL Write (DeepL SE. (2024). DeepL Write (AI writing assistant)). The text was retrieved on August 19, 2024, from https://deepl.com/write). This was done to enhance the clarity and coherence of the text. No generative AI was used. After using ChatGPT and DeepL Write, the authors reviewed and edited the content as needed and take full responsibility for the content of the publication.

## References

[1] Böstman O, Pihlajamäki H. Routine implant removal after fracture surgery: a potentially reducible consumer of hospital resources in trauma units. J Trauma. 1996;41(5):846–9. 10.1097/00005373-199611000-00013. 8913214 10.1097/00005373-199611000-00013

[2] Barcak EA, Beebe MJ, Weinlein JC. The role of Implant removal in Orthopedic Trauma. Orthop Clin North Am. 2018;49(1):45–53. 10.1016/j.ocl.2017.08.014. 29145983 10.1016/j.ocl.2017.08.014

[3] Liu L, Jian Z, Wang M, Yuan C, Li Y, Ma Y, et al. Is antibiotic prophylaxis generally safe and effective in surgical and nonsurgical scenarios? Evidence from an umbrella review of randomized controlled trials. Int J Surg. 2024;110(2):1224–33. 10.1097/js9.0000000000000923. 38016138 10.1097/JS9.0000000000000923 PMC10871558

[4] Hambrecht J, Canal C, Klingebiel F, Pfammatter C, Teuben M, Neuhaus V, et al. Elective implant removal in the upper extremity: only symptomatic patients benefit. Eur J Orthop Surg Traumatol. 2023. 10.1007/s00590-023-03777-7. 37982914 10.1007/s00590-023-03777-7 PMC10858111

[5] Vos DI, Verhofstad MH, Hanson B, van der Graaf Y, van der Werken C. Clinical outcome of implant removal after fracture healing. Design of a prospective multicentre clinical cohort study. BMC Musculoskelet Disord. 2012;13:147. 10.1186/1471-2474-13-147. 22894749 10.1186/1471-2474-13-147 PMC3493388

[6] Yao CK, Lin KC, Tarng YW, Chang WN, Renn JH. Removal of forearm plate leads to a high risk of refracture: decision regarding implant removal after fixation of the forearm and analysis of risk factors of refracture. Arch Orthop Trauma Surg. 2014;134(12):1691–7. 10.1007/s00402-014-2079-4. 25168787 10.1007/s00402-014-2079-4

[7] Langkamer VG, Ackroyd CE. Removal of forearm plates. A review of the complications. J Bone Joint Surg Br. 1990;72(4):601–4. 10.1302/0301-620x.72b4.2380210. 2380210 10.1302/0301-620X.72B4.2380210

[8] Coon D, Tuffaha S, Christensen J, Bonawitz SC. Plastic surgery and smoking: a prospective analysis of incidence, compliance, and complications. Plast Reconstr Surg. 2013;131(2):385–91. 10.1097/PRS.0b013e318277886a. 23358000 10.1097/PRS.0b013e318277886a

[9] Backes M, Schep NW, Luitse JS, Goslings JC, Schepers T. High rates of postoperative wound infection following elective Implant removal. Open Orthop J. 2015;9:418–21. 10.2174/1874325001509010418. 26401166 10.2174/1874325001509010418 PMC4578132

[10] 2018 PDSSG. 2013 PDJrMl-FrHb. Implantatentfernung nach Osteosynthese. 2018.

[11] Backes M, Dingemans SA, Dijkgraaf MGW, van den Berg HR, van Dijkman B, Hoogendoorn JM, et al. Effect of antibiotic Prophylaxis on Surgical Site infections following removal of Orthopedic implants used for treatment of Foot, Ankle, and Lower Leg fractures: a Randomized Clinical Trial. JAMA. 2017;318(24):2438–45. 10.1001/jama.2017.19343. 29279933 10.1001/jama.2017.19343 PMC5820713

[12] Sanders FRK, Penning D, Backes M, Dingemans SA, van Dieren S, Eskes AM, et al. Wound infection following implant removal of foot, ankle, lower leg or patella; a protocol for a multicenter randomized controlled trial investigating the (cost-)effectiveness of 2 g of prophylactic cefazolin compared to placebo (WIFI-2 trial). BMC Surg. 2021;21(1):69. 10.1186/s12893-020-01024-y. 33522909 10.1186/s12893-020-01024-y PMC7849087

[13] Cunha BA. Antibiotic side effects. Med Clin North Am. 2001;85(1):149–85. 10.1016/s0025-7125(05)70309-6. 11190350 10.1016/s0025-7125(05)70309-6

[14] Global burden of bacterial antimicrobial resistance. In 2019: a systematic analysis. Lancet. 2022;399(10325):629–55. 10.1016/s0140-6736(21)02724-0. 35065702 10.1016/S0140-6736(21)02724-0 PMC8841637

[15] von Elm E, Altman DG, Egger M, Pocock SJ, Gøtzsche PC, Vandenbroucke JP. The strengthening the reporting of Observational studies in Epidemiology (STROBE) statement: guidelines for reporting observational studies. Lancet. 2007;370(9596):1453–7. 10.1016/s0140-6736(07)61602-x. 18064739 10.1016/S0140-6736(07)61602-X

[16] Gupta SK, Esposito ER, Phillips R, Schwab PE, Leary EV, Hoernschemeyer DG. Effect of antibiotic prophylaxis on infection rates in Pediatric Supracondylar Humerus fractures treated with closed reduction and Percutaneous Pinning: a prospective double-blinded Randomized Controlled Trial. J Am Acad Orthop Surg. 2024;32(9):410–6. 10.5435/jaaos-d-23-00795. 38422496 10.5435/JAAOS-D-23-00795

[17] Neu HC. Cephalosporin antibiotics as applied in surgery of bones and joints. Clin Orthop Relat Res. 1984(190):50–64. 6386261

[18] Buchalter DB, Nduaguba A, Teo GM, Kugelman D, Aggarwal VK, Long WJ. Cefazolin remains the linchpin for preventing acute periprosthetic joint infection following primary total knee arthroplasty. Bone Jt Open. 2022;3(1):35–41. 10.1302/2633-1462.31.Bjo-2021-0051.R1. 35014563 10.1302/2633-1462.31.BJO-2021-0051.R1 PMC9047071

[19] Grant JM, Song WHC, Shajari S, Mak R, Meikle AT, Partovi N, et al. Safety of administering cefazolin versus other antibiotics in penicillin-allergic patients for surgical prophylaxis at a major Canadian teaching hospital. Surgery. 2021;170(3):783–9. 10.1016/j.surg.2021.03.022. 33894984 10.1016/j.surg.2021.03.022

[20] Villa JM, Pannu TS, Riesgo AM, Patel PD, Mont MA, Higuera-Rueda CA. Dual Antibiotic Prophylaxis in total knee arthroplasty: where do we stand? J Knee Surg. 2020;33(2):100–5. 10.1055/s-0039-1695742. 31470454 10.1055/s-0039-1695742

[21] Courtney PM, Melnic CM, Zimmer Z, Anari J, Lee GC. Clin Orthop Relat Res. 2015;473(7):2197–203. 10.1007/s11999-014-4062-3. 25421958 10.1007/s11999-014-4062-3 PMC4457775

[22] Gil APS, Haas OL Jr., Machado-Fernández A, Muñoz-Pereira ME, Velasques BD, da Rosa BM, et al. Antibiotic prophylaxis in orthognathic surgery: an overview of systematic reviews. Br J Oral Maxillofac Surg. 2021;59(10):1174–85. 10.1016/j.bjoms.2021.05.010. 34465488 10.1016/j.bjoms.2021.05.010

[23] Madaan A, Li JT. Cephalosporin allergy. Immunol Allergy Clin North Am. 2004;24(3):463–76. 10.1016/j.iac.2004.03.009. vi-vii. 15242721 10.1016/j.iac.2004.03.009

[24] Redais C, Murison JC, Bazile F, de L’Escalopier N, Grosset A. Preoperative antibiotics reduce early surgical site infections after orthopaedic implant removal: a propensity-matched cohort study. J Hosp Infect. 2024;143:18–24. 10.1016/j.jhin.2023.10.013. 38511861 10.1016/j.jhin.2023.10.013

[25] Shao J, Zhang H, Yin B, Li J, Zhu Y, Zhang Y. Risk factors for surgical site infection following operative treatment of ankle fractures: a systematic review and meta-analysis. Int J Surg. 2018;56:124–32. 10.1016/j.ijsu.2018.06.018. 29929022 10.1016/j.ijsu.2018.06.018

[26] Mielke M, Hansis M. Prevention of Surgical Site infections: current recommendation of the German Commission of Hospital Hygiene and Infection Prevention. Bundesgesundheitsblatt Gesundheitsforschung Gesundheitsschutz. 2018;61(4):371–3. 10.1007/s00103-018-2721-3. „Prävention postoperativer Wundinfektionen: Aktualisierte Empfehlung der Kommission für Krankenhaushygiene und Infektionsprävention (KRINKO). 29536112 10.1007/s00103-018-2721-3

[27] Brimmo AT, Glia A, Barajas-Gamboa JS, Abril C, Rodríguez J, Kroh M, et al. Ventilation-based strategy to manage intraoperative aerosol viral transmission in the era of SARS-CoV-2. Life (Basel). 2024;14(3). 10.3390/life14030313. 10.3390/life14030313 PMC10970813 38541639

[28] Rather IIG, Shafiq N, Pandey AK, Bhandari RK, Malhotra S, Chouhan DK. Antibiotic Prophylaxis for Orthopaedic Implant removal: what does the evidence say? Curr Drug Saf. 2023;18(1):116–20. 10.2174/1574886317666220429081207. 36748234 10.2174/1574886317666220429081207

[29] Suda AJ, Heilgeist E, Tinelli M, Bischel OE. High early post-operative complication rate after elective aseptic orthopedic implant removal of upper and lower limb. J Orthop Res. 2018;36(3):1035–9. 10.1002/jor.23718. 28862357 10.1002/jor.23718

[30] Dong X. Surgical site infection in upper extremity fracture: incidence and prognostic risk factors. Med (Baltim). 2022;101(35):e30460. 10.1097/md.0000000000030460. 10.1097/MD.0000000000030460 PMC9439831 36107575

[31] Emery CF, Kiecolt-Glaser JK, Glaser R, Malarkey WB, Frid DJ. Exercise accelerates wound healing among healthy older adults: a preliminary investigation. J Gerontol Biol Sci Med Sci. 2005;60(11):1432–6. 10.1093/gerona/60.11.1432. 10.1093/gerona/60.11.1432 16339330

[32] Song L. Effects of Exercise or Mechanical Stimulation on Bone Development and Bone Repair. Stem Cells Int. 2022;2022:5372229. 10.1155/2022/5372229. 36213684 10.1155/2022/5372229 PMC9534715

[33] Eberhardt RT, Raffetto JD. Chronic venous insufficiency. Circulation. 2014;130(4):333–. 10.1161/circulationaha.113.006898. 46. 25047584 10.1161/CIRCULATIONAHA.113.006898

